# Purification of recombinant IpaJ to develop an indirect ELISA-based method for detecting *Salmonella enterica* serovar Pullorum infections in chickens

**DOI:** 10.1186/s12917-018-1753-0

**Published:** 2019-01-03

**Authors:** Qiuchun Li, Yue Zhu, Kequan Yin, Lijuan Xu, Chao Yin, Yang Li, Jingwei Ren, Yu Yuan, Xinan Jiao

**Affiliations:** 1 Key Laboratory of Prevention and Control of Biological Hazard Factors (Animal Origin) for Agri-food Safety and Quality, Ministry of Agriculture of China, Yangzhou University, Yangzhou, China; 2 Jiangsu Key Laboratory of Zoonosis/Jiangsu Co-Innovation Center for Prevention and Control of Important Animal Infectious Diseases and Zoonoses, Yangzhou University, Yangzhou, China; 3 Joint International Research Laboratory of Agriculture and Agri-Product Safety of Ministry of Education of China, Yangzhou University, Yangzhou, China

**Keywords:** *Salmonella enterica* serovar Pullorum, ELISA, IpaJ

## Abstract

**Background:**

*Salmonella enterica* serovar Pullorum is a host-restricted serotype causing infection in poultry. The pathogen can not only cause acute infection in young chicks with high mortality and morbidity, but also persist in adult chickens without evident clinical symptoms and lead to vertical transmission. To eradicate *S.* Pullorum in poultry farms, it is necessary to establish an efficient method to monitor the prevalence of the pathogen in adult chickens. The protein IpaJ is a specific immunogen in *S.* Pullorum and is not detected in closely related serotypes, such as *S.* Gallinarum and *S.* Enteritidis.

**Results:**

In the present study, IpaJ was expressed as a recombinant fusion protein MBP-IpaJ in *E. coli*. The purified MBP-IpaJ was used as a coating antigen to develop an indirect ELISA assay, which was applied to the detection of *S.* Pullorum infection in chickens. The indirect ELISA assay demonstrated that antibodies produced against IpaJ were detectable in antisera of chickens infected with *S.* Pullorum in the second week, stably increased until the tenth week, and persisted at a high level in the following two weeks. Furthermore, the ELISA method detected four positive samples out of 200 clinical antiserum samples collected from a poultry farm, and the positive samples were confirmed to be reacted with *S.* Pullorum using the standard plate agglutination test.

**Conclusions:**

The established indirect ELISA using the IpaJ protein is a novel method for specific detection of *S*. Pullorum infection, and contribute to eradication of pullorum disease in the poultry industry.

**Electronic supplementary material:**

The online version of this article (10.1186/s12917-018-1753-0) contains supplementary material, which is available to authorized users.

## Background

Pullorum disease remains a problem in the poultry industry in developing countries such as China and Brazil. The causative agent is *Salmonella enterica* serovar Pullorum (*S.* Pullorum), a host-restricted serotype mainly infecting poultry [1]. Because *S.* Pullorum persistently exists in infected adult chickens, the pathogen can be transmitted vertically to offspring. Therefore, to reduce the prevalence of the disease in poultry farms, efficient surveillance has become increasingly important and essential. Although bacterial culture is the standard method for monitoring *Salmonella* infection due to the accuracy of the testing method, culture-based procedure for *Salmonella* isolation and serotype identification are labor intensive, expensive to perform, and time-consuming, thereby limiting its application in clinical diagnosis [2–5]. Thus, it is necessary to develop new methods appropriate to specifically monitoring the prevalence of *S.* Pullorum in poultry farms.

Serology detection has frequently been used to identify infected and carrier animals in both surveillance and epidemiological studies [6]. The gold standard serological *Salmonella* detection method in chickens is the agglutination test, which played a significant role in eradicating *S*. Pullorum in Europe [7]. However, the agglutination test mainly uses membrane-bound antigens of *Salmonella*, and cannot differentiate *S*. Pullorum from *S*. Gallinarum and *S*. Enteritidis because of the shared O9 antigen in the three serotypes [8]. ELISA methods were subsequently developed to detect antibodies against LPS or FliC in avian sera [8–11], but problems of cross-reactivity could not be resolved because of antigen conservation in the different serotypes. Therefore, developing an ELISA assay specific to *S*. Pullorum infection is useful for surveillance in the poultry industry.

The IpaJ protein is a new antigen reported to be specific to *S.* Pullorum, and not detected in *S.* Gallinarum and *S.* Enteritidis. Our previous studies revealed that > 99% *S.* Pullorum isolates carry the *ipaJ* gene, which is expressed during *S.* Pullorum infection [12–14]. In the present study, the *ipaJ* gene was cloned into the bacterial expression vector pMBL-c5x and expressed in *E. coli.* Using the purified MBP-IpaJ protein, an indirect ELISA method was developed and applied to sera from *S*. Pullorum-infected chickens. The method has the potential to contribute to rapid and specific diagnosis of *S.* Pullorum infection in chickens.

## Results

### Expression, purification, and detection of recombinant MBP-IpaJ

**Fig. 1:**
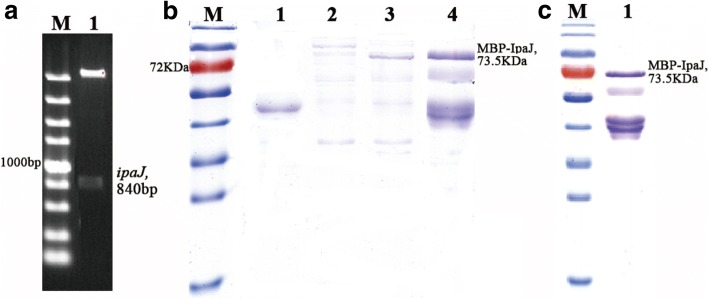
Expression and purification of recombinant MBP-IpaJ. (**a**) Amplified *ipaJ* product for construction of the prokaryotic recombinant plasmid pMAL-c5X-*ipaJ*. Lane 1: plasmid pMAL-c5X-*ipaJ* digested with *Nde*I and *Eco*RI (Takara, Japan). (**b**) Expression of recombinant protein MBP-IpaJ. Lane M: the size of protein ladders (kDa) shown in the figure are 130, 95, 72 (red), 55, 43, 34, 26, and 17, respectively. Lane 1: induced ER2523-pMAL-c5X as negative control; Lane 2: the supernatant of non-induced ER2523-pMAL-c5X-*ipaJ*; Lane 3: the supernatants of ER2523-pMAL-c5X-*ipaJ* induced at 30 °C for 4 h; Lane 4: the supernatants of ER2523-pMAL-c5X-*ipaJ* induced at 37 °C for 4 h. (**c**) purified recombinant MBP-IpaJ

The *ipaJ* gene, 840 bp in length, was successfully amplified from the strain C79–13 and cloned into the prokaryotic vector pMAL-c5X to create pMAL-c5X-*ipaJ*. The sequence of *ipaJ* was identified via sequencing analysis and restriction digestion (Fig. 1a).

The recombinant strain ER2523-pMAL-c5X-*ipaJ* was subjected to induction with 0.3 mM IPTG, and SDS-PAGE results showed that MBP-IpaJ was successfully expressed in the host cells (Fig. 1b). The molecular weight of MBP-IpaJ was estimated to be approximately 73.5 kDa. Additionally, the MBP-IpaJ fusion protein was expressed in the supernatant and successfully purified using affinity chromatography (Fig. 1c).

Using ER2523-pMAL-c5X-*ipaJ*, both of the MBP and MBP-IpaJ fusion proteins were expressed and purified using the amylose column. Western blot analysis detected two bands representing MBP and MBP-IpaJ, respectively (Additional file 1: figure S1). In addition, because MBP is a tag aiding the correct folding of the fusion protein, MBP-IpaJ was detected in the supernatants of the lysates (Fig. 1b).

### Establishment and specificity of the indirect ELISA assay

**Fig. 2:**
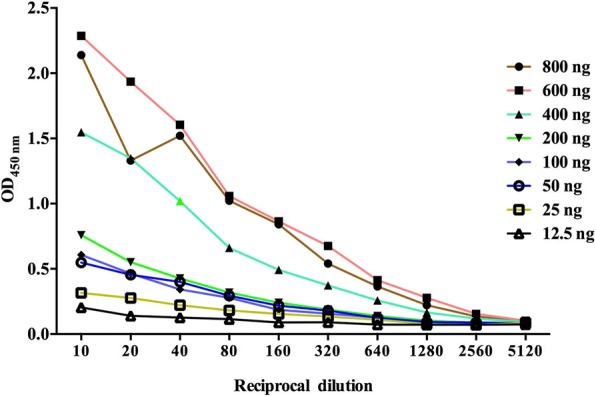
Checkerboard for optimization of the indirect ELISA assay using the recombinant MBP-IpaJ. The coating antigen MBP-IpaJ was added into the well of the ELISA plate at concentrations of 800 ng, 400 ng, 200 ng, 100 ng, 50 ng, 25 ng, and 12.5 ng. Hundred microliters of the antisera used was added per well with dilutions from from 1:10 to 1:5120. The inflection point at 400 ng of MBP-IpaJ and 1:40 dilution of antiserum was considered to represent the optimum concentrations

The matrix method was used to determine the concentration of the coating protein MBP-IpaJ. When the concentration of protein was 400 ng, the OD450 reached 1.02 at a 1:40 dilution ratio of antiserum (Fig. 2). Checkerboard titrations indicated that 400 ng of coating protein and a 1:40 dilution of chicken antiserum should be used in the indirect ELISA assay.

**Fig. 3:**
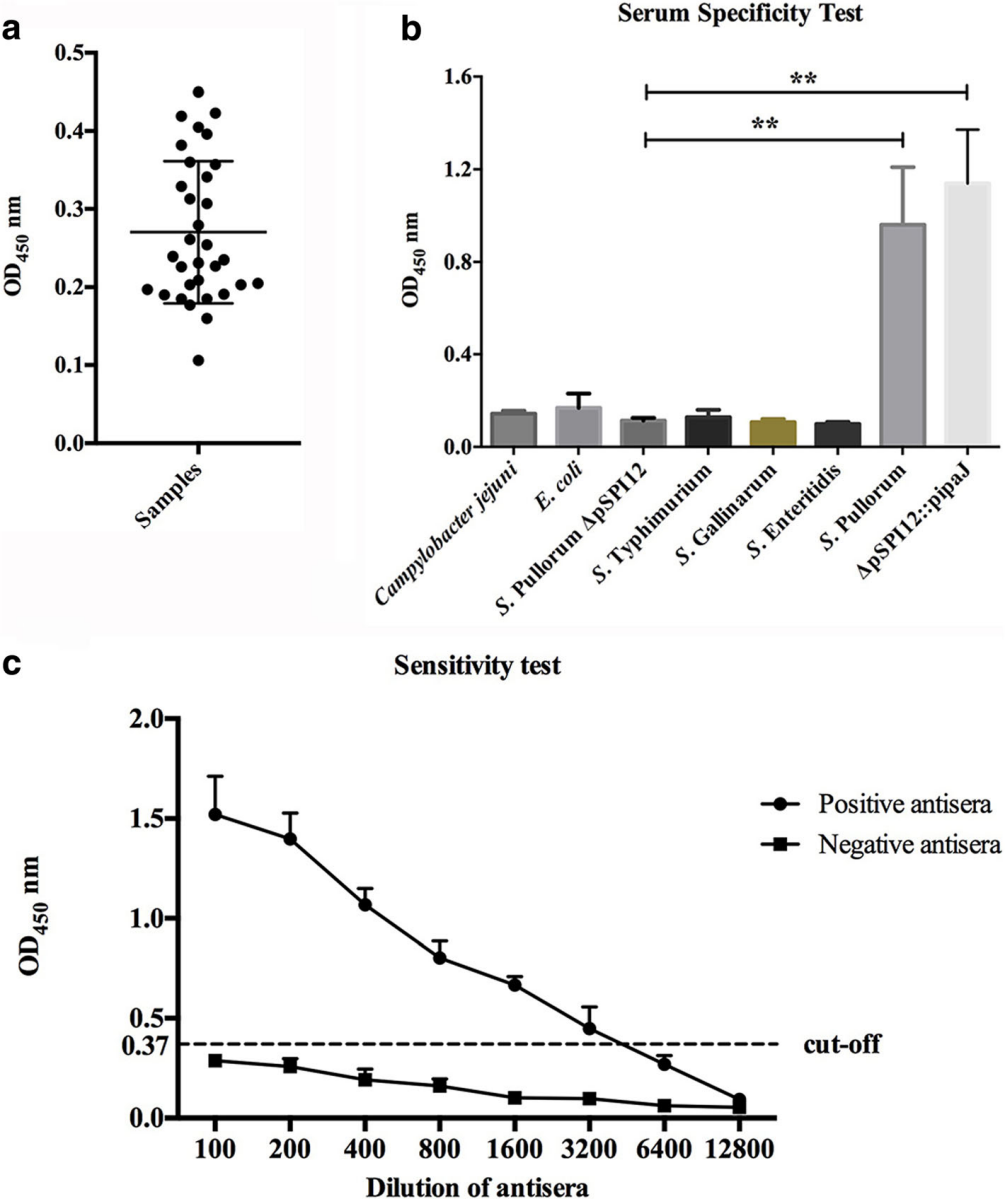
Determination of the cut-off value, specificity, and sensitivity of the indirect ELISA assay. (**a**) 32 antisera samples from chicken not exposed to *S*. Pullorum were subjected to the indirect ELISA assay to obtain the cut-off value. (**b**) Antisera samples from chicken infected with other *Salmonella* serotypes or avian bacteria (*E. coli* and *Campylobacter jejuni*) were used to determine the specificity of the indirect ELISA assay. All of the OD_450nm_ values of these antisera samples were below 0.4. (**c**) Positive antisera against *S.* Pullorum were serially diluted from 1:100 to 1:12800, and then used to identify the sensitivity of the indirect ELISA assay (**: *p* < 0.01)

The 32 antisera from chickens not exposed to *S.* Pullorum was used to determine the cut-off value. The cut-off value for positive results was determined to be 0.45 based on the mean OD450 and SD values obtained from these 32 *S.* Pullorum-negative antisera (Fig. 3a). Specificity evaluation of the indirect ELISA assay demonstrated that the OD450 values of all of the antisera from chickens infected with other *Salmonella* serotypes or microbial strains were below the cut-off value (Fig. 3b). Additionally, the indirect ELISA method could differentiate antisera from *ipaJ*-deleted *S.* Pullorum(∆pSPI12)-infected chickens with that from chickens infected with *ipaJ*-positive *S.* Pullorum(∆pSPI12::p*ipaJ*) (Fig. 3b). Sensitivity test was performed using antisera from chickens infected with *S.* Pullorum two weeks post-infection. As shown in Fig. 3c, the detection limit for the indirect ELISA method was 1:3200 dilution of the *S.* Pullorum-positive antisera.

### The development of antibodies against IpaJ in *S.* Pullorum-infected chickens

**Fig. 4:**
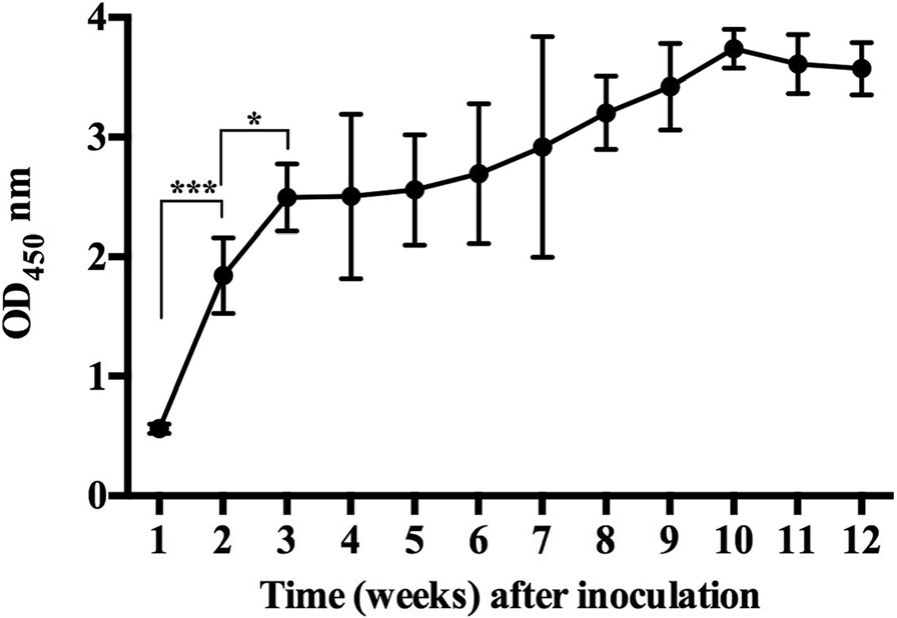
Changes in levels of antibodies against IpaJ in antiserum from *S.* Pullorum-infected chickens. Antiserum samples from ten *S.* Pullorum infected chicken were subjected to the detection of antibodies against IpaJ using the established indirect ELISA assay. The antisera were collected from the first week after inoculation to the twelfth week. (*: *p* < 0.05; **: *p* < 0.01; ***: *p* < 0.001)

The indirect ELISA assay was used to detect the changes in levels of antibodies against IpaJ in antiserum from ten *S.* Pullorum-infected chickens during 12 successive weeks. As shown in Fig. 4, the antibodies against IpaJ were not detected in the first week, but increased dramatically in the second week as well as the third week. From the third week to the tenth week, the antibodies against IpaJ showed a stable increase. In the eleventh and twelfth weeks, the antibodies remained at a high level.

### Application of the indirect ELISA assay in clinical samples

**Fig. 5:**
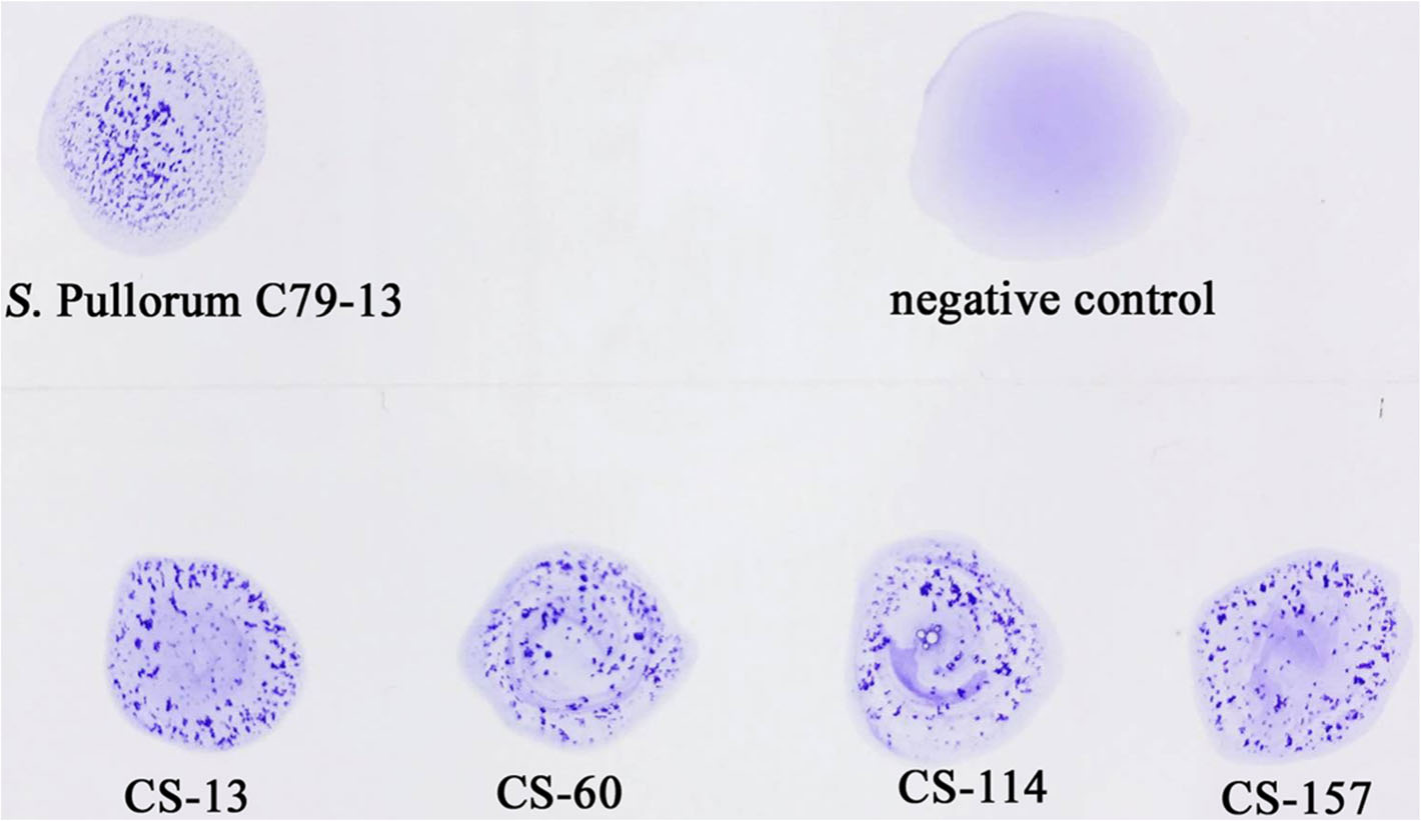
The application of the indirect ELISA assay in clinical antisera samples. Two hundred chicken antisera samples from a poultry farm were collected and subjected to the indirect ELISA assay. Four samples with OD450 > 0.45 were considered to be positive samples. The four positive antisera identified were subjected to the PAT test with *S.* Pullorum strain C79–13

The indirect ELISA assay was used to test 200 clinical antisera collected from a poultry farm, and the result showed that four out of the 200 samples were positive for antibodies against IpaJ. No specific method to detect antisera from *S*. Pullorum has been reported so far; the plate agglutination test (PAT) was used to confirm the reaction of these positive antisera with *S.* Pullorum strain C79–13 (Fig. 5). However, the PAT could not differentiate the antisera of *S.* Pullorum-infected chickens from those of chicken infected with *S.* Gallinarum and/or *S.* Enteritidis carrying the same O9 antigen.

## Discussion

Isolation of bacteria from infected animals is considered as the gold standard for *Salmonella* diagnosis due to its accuracy, but the problems of significant time consumption and complexity limits its clinical application [4]. Serological tests for *Salmonella* infection have been widely used in clinical diagnosis and epidemic analysis due to their convenience, sensitivity, and relatively low cost [15, 16]. In the present study, we developed an indirect ELISA method to detect antibodies against IpaJ protein to identify *S.* Pullorum infections in poultry. IpaJ is a specific antigen identified in *S.* Pullorum, and PCR analysis of the *ipaJ* gene could be used to differentiate *S.* Pullorum from the other genetically related serotypes, such as *S.* Gallinarum and *S.* Enteritidis [13]. In addition, the protein is an immunogen inducing chickens to produce antibodies [17]. Specificity analysis of our ELISA method confirmed that antibodies against IpaJ could be detected in *S.* Pullorum-infected chickens, but not be detected in *S*. Gallinarum and *S.* Enteritidis-infected chickens.

In the present study, the indirect ELISA method revealed that chickens infected by *S.* Pullorum could produce high titers of antibodies against IpaJ, which persisted for more than eleven weeks. These results support the utility of IpaJ antibody detection for specific identification of *S.* Pullorum infections. Although the official standard in China for serological detection of pullorum disease in chickens is the plate agglutination test (PAT), this test cannot identify serotypes of *Salmonella*, with interpretation of the results being somewhat subjective and prone to error [7]. In the present study, four out of 200 chicken antisera samples were detected to have *S.* Pullorum infection, while the PAT results confirmed that these antisera could react with *S*. Pullorum, *S*. Gallinarum and *S*. Enteritidis. These findings demonstrated that the specificity of the indirect ELISA method was better than that of PAT. However, MBP fusion protein, which was used as the coating antigen could also react with antibodies against MBP, and such antibodies have been found to have minor seroreactivity [18]. MBP-tagged avian influenza M2 protein has been developed as a coating antigen in the M2e-MBP ELISA method for differentiating avian influenza infected chickens from vaccinated ones [19]. The indirect ELISA method developed in the present study requires optimization in field applications. Further studies will be done to test a larger number of antisera samples from chickens infected with *S.* Pullorum or other *Salmonella* serotypes, and assess the specificity of the method.

## Conclusions

In summary, the present study developed an indirect ELISA method for identifying *S.* Pullorum infection in chickens based on detection of antibodies against IpaJ protein. The method can not only differentiate pullorum disease from other avian *Salmonellosis*, but can also serve as a novel method for monitoring the *S*. Pullorum infection and contribute to eradication of pullorum disease in the poultry industry.

## Methods

### Bacterial strains and growth conditions

The *S.* Pullorum reference strain C79–13 obtained from the China Institute of Veterinary Drug Control was used in this study. The *ipaJ* deleted strain ∆pSPI12 and complementary strain ∆pSPI12::p*ipaJ* were preserved in our lab (14). The prokaryotic expression vector pMAL-c5X (NEB, USA) was used to construct recombinant expression plasmids in the host strain *E. coli* ER2523 (NEB, USA). Bacteria with recombinant plasmids were grown in Luria Bertani (LB) broth containing ampicillin (100 μg/ml).

### Construction of recombinant expression plasmid

Using the published *ipaJ* gene sequence (Accession Number: GU949535) in GenBank, the primes pMAL-c5X-*ipaJ*-F/R (c5X-*ipaJ*-F: GAGGGAAGGATTTCACATATGCGGTTAAAATTTATCAG; c5X-*ipaJ*-F: TATTTAATTACCTGCAGGGAATTCTCAAGCTGACAAGACAATAGA) were designed to amplify the gene from the strain C79–13 using PCR. PCR analysis was performed in a 50 μl volume containing 2.5 U of Taq DNA polymerase, 10 μl 5 x SF buffer, 200 μM dNTP mix, 0.4 μM upstream and downstream primers, and 1 μl DNA template. PCR amplification was done with a pre-denaturation step at 95 °C for 5 min followed by 30 cycles of 95 °C for 50 s, 61 °C for 50 s, and 72 °C for 1 min, and the reaction terminated with a final extension at 72 °C for 10 min. The PCR products were then resolved in a 1% agarose gel after electrophoresis and purified using the MiniBEST Agarose Gel DNA Extraction kit (Takara, Japan). The purified *ipaJ* PCR products were then ligated to pMAL-c5X vector. After transformation of the ligated products into the ER2523 competent cells, the colonies containing the recombinant plasmid were subject to PCR identification and sequencing analysis. The recombinant bacterial strain carrying the pMAL-c5X-*ipaJ* plasmid was named ER2523-pMAL-c5X-*ipaJ*.

### Expression and purification of recombinant MBP-IpaJ

An overnight culture of the recombinant strain ER2523-pMAL-c5X-*ipaJ* was inoculated into fresh LB medium with ampicillin at a 1:100 dilution. When the OD600 reached 0.4–0.6, IPTG was added to the medium with at a final concentration of 0.3 mM to induce protein expression, and the bacteria cultured at 37 °C for 4 h at a shaking speed of 180 rpm. The bacterial pellets were collected for ultrasonic lysis, the supernatants of lysed bacterial cultures containing the MBP-IpaJ protein were subjected to SDS-PAGE and stained with 0.025% coommassie brilliant blue R-250 (Sigma, USA). Purification of the protein was performed following the manufacture’s instruction by using the pMAL™ Protein Fusion and Purification System (NEB, USA).

### Western blot analysis

The cell lysates or purified recombinant proteins were subjected to SDS-PAGE and transferred to NC membrane using a Pyxis™ Gel Processor (Pyxis, China). The NC membrane was blocked in 5% BSA and then incubated in anti-MBP antibodies at a 1:8000 dilution, or anti-serum against IpaJ at a 1:1000 dilution for 2 h at 37 °C. After incubation with goat anti-mouse IgG-HRP at a 1:10000 dilution for 1 h, the NC membrane was stained with an ECL chromogenic kit (Thermo, USA) and scanned by using an Amersham Imager 600 imagers (GE Healthcare, USA).

### Establishment of an indirect ELISA assay

The recombinant MBP-IpaJ was used as the coating protein in the ELISA assay. To determine the best concentration of the coating protein, MBP-IpaJ was diluted to the following concentrations: 8 μg/ml, 6 μg/ml, 4 μg/ml, 2 μg/ml, 1 μg/ml, 0.5 μg/ml, 0.25 μg/ml, and 0.125 μg/ml. Hundred microliters per well of each dilution was then added to 12 wells of the ELISA plate, and incubated at 4 °C for 14–16 h. After washing with PBST three times, the plate was blocked with 200 μl per well of 1% BSA in PBS at 37 °C for 2 h. Antisera from *S.* Pullorum-infected chickens were diluted from 1:10 to 1:5120, and 100 μl per well of each dilution was added to the plate and incubated at 37 °C for 2 h. Following incubation with 100 μl per well of 1:10000 rabbit anti-chicken IgG-HRP at 37 °C for 1 h, the plate was stained with TMB for 10 min at 37 °C and the reaction terminated with 2 M H_2_SO_4_. The OD450 value was measured to determine the best concentration of MBP-IpaJ and the appropriate dilution of the antiserum.

### Specificity and sensitivity test

The specificity of the indirect ELISA assay was evaluated using other avian *Salmonella* serotypes including *S.* Gallinarum, *S.* Enteritidis, *S.* Typhimurium, and other poultry bacteria, including *E. coli*, and *Campylobacter jejuni*. The antisera from chicken infected with these bacteria were collected and subjected to the indirect ELISA assay to evaluate the specificity of the method. The sensitivity test of the indirect ELISA method was performed using positive antisera against *S.* Pullorum from infected chickens. The antisera were serially diluted from 1:100 to 1:12800 to be subjected to the indirect ELISA analysis, while the corresponding diluted antisera from non-infected SPF chickens were used as the negative controls.

### The developmental of antibodies against IpaJ in vivo

To evaluate the indirect ELISA assay for the detection of *S.* Pullorum infection, the production of antibodies against IpaJ during *S.* Pullorum infection in SPF chickens (Beijing Merial Vital Laboratory Animal Technology Co., Ltd., China) was monitored. All of one-week old female SPF chickens were given antibiotic-free feed and water. The animal experiments were undertaken with the permission of the Animal Care and Ethics Committee of Yangzhou University. The *S.* Pullorum reference strain C79–13 was inoculated intramuscularly (i.m.) to ten one-week old SPF chickens with 1 × 10^6^ CFU per chicken. The antiserum was collected every week during the following 12 weeks. The collected antisera were preserved at − 70 °C, and then subjected to the indirect ELISA assay.

### Application of the ELISA method to clinical samples

Two hundred antiserum samples were collected from a poultry farm. All of the samples were monitored using the indirect ELISA assay and the results compared with those of the PAT method used to detect antisera from chickens infected with *S.* Pullorum and *S.* Gallinarum [1].

### Statistical analysis

The data was analyzed using the Student’s *t*-test with pairwise analysis. A *p* < 0.05 was considered as statistically significant. All analyses were performed in the software GraphPad Prism version 7.

## Additional file

Additional file 1: Identification of recombinant MBP-IpaJ protein via Western blot analysis. The immunoblotting assay was used to detect the expression of MBP-IpaJ protein using anti-MBP antibody recognizing the tag. Two bands were detected and found to represent MBP-IpaJ (73.5 kDa) and MBP (42 kDa). (DOCX 148 kb)

## Abbreviations

ELISA: Enzyme-linked immunosorbent assay
i.m.: intramuscularly
LB: Luria Bertani
LPS: lipopolysaccharide
MBP: Maltose binding protein
PAT: Plate agglutination test
*S*. Enteritidis: *Salmonella enterica* serovar Enteritidis
*S*. Gallinarum: *Salmonella enterica* serovar Gallinarum
*S.* Pullorum: *Salmonella enterica* serovar Pullorum
*S.* Typhimurium: *Salmonella enterica* serovar Typhimurium
SDS-PAGE: Sodium dodecyl sulfate-polyacrylamide gel electrophoresis.
SPF: Specific pathogen free
TMB: Tetramethylbenzidine
