# Supplementary material for: Purification of recombinant IpaJ to develop an indirect ELISA-based method for detecting Salmonella enterica serovar Pullorum infections in chickens
Source: BMC Vet Res. 2019 Jan 3;15:3. doi: 10.1186/s12917-018-1753-0 (PMC6318851; doi:10.1186/s12917-018-1753-0)
Supplement: Supplementary file 1 — Identification of recombinant MBP-IpaJ protein via Western blot analysis. The immunoblotting assay was used to detect the expression of MBP-IpaJ protein using anti-MBP antibody recognizing the tag. Two bands were detected and found to represent MBP-IpaJ (73.5 kDa) and MBP (42 kDa). (DOCX 148 kb) [file 12917_2018_1753_MOESM1_ESM.docx]

**Additional Fig. S1**

**
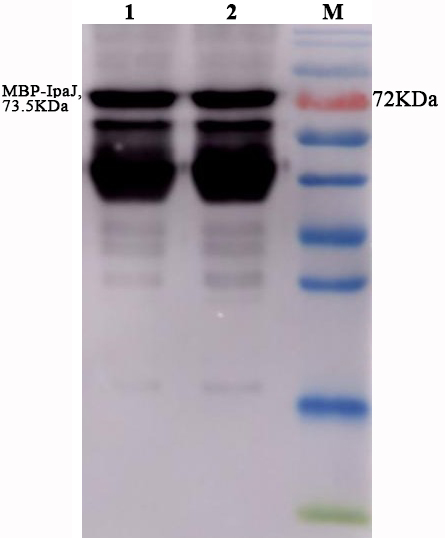
**

Fig S1. Identification of recombinant MBP-IpaJ protein via Western blot analysis. The immunoblotting assay was used to detect the expression of MBP-IpaJ protein using anti-MBP antibody recognizing the tag. Two bands were detected and found to represent MBP-IpaJ (73.5 kDa) and MBP (42 kDa).
